# Beyond the algorithm: embedding ethics for trustworthy AI in radiology and oncology

**DOI:** 10.3389/fdgth.2026.1756256

**Published:** 2026-04-20

**Authors:** Mónica Cano Abadía, Melanie Goisauf

**Affiliations:** BBMRI-ERIC, ELSI Services and Research, Graz, Austria

**Keywords:** AI ethics, embedded ethics, interdisciplinarity, radiology, stakeholder engagement, trustworthy AI

## Abstract

**Background:**

Artificial intelligence (AI) in radiology and oncology promises improvements in diagnostic accuracy and efficiency yet introduces complex ethical and societal challenges. Governance efforts frequently rely on high-level principles such as trustworthiness and fairness, which risk becoming ineffective when not grounded in specific contexts. This study presents findings from our work on ethical and societal aspects of AI within the EuCanImage project.

**Methods:**

We conducted a multi-method empirical study involving literature reviews, interviews, and workshops with developers, clinicians, and other stakeholders. The study explored how ethical concerns emerge in real-world settings and how they are shaped by institutional, clinical, and sociotechnical dynamics.

**Results:**

Findings indicate that ongoing interdisciplinary involvement is essential to address explainability, accountability, bias, and social impact in radiological AI. The literature review identified four guiding dimensions of trustworthy AI (i.e., explainability and interpretability, trust and trustworthiness, responsibility and accountability, and justice and fairness) which remain difficult to operationalize without concrete procedural guidance. Empirical findings highlight that ethical issues cannot be addressed solely as technical problems or abstract principles. Trustworthiness emerged as relational and co-constructed through interactions among very diverse stakeholders.

**Conclusion:**

We propose a structured, multi-stakeholder AI development pathway that advances from decontextualized, principle-driven ethics toward embedded, interdisciplinary approaches attentive to clinical realities, power relations, and socio-cultural conditions, by strengthening stakeholder engagement for trustworthy AI in cancer care.

## 1 Introduction

Artificial intelligence (AI) is rapidly entering clinical practice, especially in diagnostic imaging and decision support. As AI systems become more integrated into care pathways, trustworthiness has emerged as a central concept to foster acceptance and responsible use. Based on key ethical values such as respect, autonomy, beneficence, and justice (1), many high-level guiding principles and recommendations have been formulated to tackle these issues. Such principles and recommendations have also been communicated at the level of the European Union (2) and in initiatives such as FUTURE-AI (3). Governance efforts frequently rely on high-level principles such as trustworthiness and fairness, which risk becoming ineffective when not grounded in specific clinical, technological, and social contexts. Previous research (4) emphasized users' preference for practical forms of intelligibility that support clinical judgment and accountability over detailed technical details. This indicates that trust in AI develops within existing professional practices rather than replacing them, underscoring the need to embed trust and ethics throughout the entire AI development and implementation process.

Within radiology and oncology, where decisions may affect diagnosis, treatment trajectories, and patient wellbeing, ensuring that AI systems are trustworthy is particularly important. Accuracy and efficiency contrast with concerns regarding opacity, bias, and responsibilities among healthcare professionals. Successful implementation is therefore not only a technical question, but also about clinical workflows, institutional structures, professional experiences, and patient expectations.

While the term “trustworthy AI” has been subject to rightful philosophical criticism (5, 6), as only humans, and not machines, can be (un)trustworthy, this terminology has crystallized in the discourse of ethics AI; by keeping using the term we aim at reframing the concept in a more nuanced, empirically-informed and ethically-grounded way that makes justice to the complexity of this topic.

A comprehensive approach to trustworthy AI must therefore go beyond the algorithm. We should strive to go from principle-driven abstractions to more situated approaches that account for real-world practices of development, evaluation, and use. Embedding ethics and Social Sciences and Humanities (SSH) research into the lifecycle of AI for imaging requires interdisciplinary attention to multilayered aspects, such as everyday decision-making, communication, interprofessional collaboration, and the normative assumptions built into data, models, and deployment strategies. This approach would recognize AI not as a neutral tool but as a socio-technical actor that shapes and, at the same time, is shaped by clinical environments.

This article discusses the results of an analysis conducted on the ethical and societal implications of AI in oncologic imaging, utilizing empirical data gathered within the EuCanImage project. The goal of EuCanImage was to build a FAIR (Findable, Accessible, Interoperable, Reusable) cancer imaging platform, while integrating ethical, legal, and social/societal implications (ELSI) through comprehensive empirical research including literature reviews, qualitative interviews, and stakeholder workshops. By exploring how key stakeholders discuss trust, accountability, and practical intelligibility, we aim to suggest a pathway for more ethically grounded AI.

This paper is empirically grounded but also conceptually oriented: we use findings from literature synthesis and interviews to develop an empirically informed argument for embedding ethical and social-science expertise into the AI lifecycle in radiology/oncology. Our aim is not to “prove” embedded ethics as the only solution, but to show how the challenges described by stakeholders and the literature motivate a shift from principle-level guidance to situated, interdisciplinary practices.

This article proceeds as follows: first, we outline the methodological grounding of our multi-method study; second, we synthesize the literature on the four guiding dimensions of trustworthy AI, highlighting trust and trustworthiness as integrative concepts; third, we present empirical findings from expert interviews to examine how trustworthiness is constructed in practice; finally, we propose an embedded ethics pathway informed by both the literature and empirical analysis.

## 2 Materials and methods

To investigate the ethical and social implications of AI in radiology, we designed a multi-method empirical study combining systematic literature reviews, qualitative interviews, and expert engagement. The study protocol received ethics approval from the Ethics Committee of the Medical University of Graz (EK-Number 33-650 ex 20/21).

### 2.1 Systematic literature review

In the first phase, we conducted a comprehensive review of ethical and societal issues associated with AI in radiology and of how these issues have been addressed in the field. A systematic search of state-of-the art academic literature between July and December 2021 was performed using five databases (Google Scholar, Microsoft Academic, PubMed, Scopus, and Web of Science). Twelve search strings were created that included terms relevant to the topic under study (e.g., “AI,” “ethics,” “radiology,” “imaging,” “oncology,” “cancer,” “predictive medicine,” “trustworthy,” “explainable,” “black box,” and “breast cancer”; “IT” was defined as an exclusion criterion to refine the search and limit it to the ethical and societal aspects related to AI). The search focused on articles written in English language and to papers published after 2017. Outputs consisted mainly of peer-reviewed journal articles, but also included literature in the form of commentaries, reports, and book chapters. Following the screening of relevant records, the full texts of eligible publications (*n* = 56) were analyzed using thematic analysis (7). Each article was openly coded to identify overarching themes, patterns, and conceptual gaps, as well as to assess the depth and scope of ethics-related discussions. Findings of this initial review have been published in Goisauf and Cano Abadía (8).

To update this evidence base for this article, we conducted a rapid review following systematic review principles, including transparent documentation of selections steps based on PRISMA (9) (see Supplementary Figure S1 in Supplementary Material) and explicit acknowledgement of inclusion and exclusion criteria to support reliability and credibility. Based on the initial literature review, the updated rapid review was structured around the key themes identified in the original review (“AI”, “ethics”, “radiology”, “black box”, “bias”, “trust”, “trustworthy”, “explainability”, “interpretability”, “accountability”, and “responsibility”; see Supplementary Table S1 in Supplementary Material) and incorporated newly published evidence to update and refine the findings. Searches were carried out in Google Scholar, PubMed, and Scopus, targeting peer-reviewed, open-access, full-text articles published in English between January 2023 and August 2024. The updated rapid review was conducted using three databases instead of the five used in the initial search, as Microsoft Academic has been discontinued and institutional access to Web of Science was not available at the time of the update. Given the volume of results, the top ten outputs from each search were screened. Inclusion criteria required that studies substantively address ethical and/or societal implications of AI in radiology. Exclusion criteria were: (1) technical-only papers without ethical or societal considerations; (2) papers addressing explainability or related issues solely in technical terms without engaging ethical or social dimensions; and (3) studies focused on highly specific medical applications. After screening, 42 articles met these criteria and were subject to thematic analysis following the same analytic framework used in the initial review.

### 2.2 Qualitative expert interviews

We conducted thirteen semi-structured interviews with experts from the EuCanImage consortium and beyond. Eleven interviews took place face-to-face, and two interviews were conducted via Zoom. The interviews were conducted based on a topic guide that covered work practices and areas of expertise as well as attitudes towards the role, benefits and limits of radiological AI. Interview partners were AI and platform developers, clinicians, and patient representatives. All interviews were audio-recorded, transcribed, and analyzed in-depth using the Atlas.ti software, drawing on open coding and analytic strategies from Grounded Theory methodology (10).

Although the topic guide covered work practices, expertise, and attitudes toward AI, the interviews were analyzed inductively using open coding in line with Grounded Theory methodology. The four dimensions identified in the literature review did not predetermine the coding framework. Rather, the empirical analysis allowed themes to emerge from the material. Trust and trustworthiness emerged as central organizing themes across stakeholder accounts, which is why they receive particular analytical attention in the empirical section.

### 2.3 Expert workshop

Building on insights from the literature and interviews, we convened an expert workshop on the ethical and social implications of AI in cancer-related biomedical research (Berlin, 5–6 May 2022). Experts discussed topics such embedding ethics and SSH research into the development of AI, algorithmic impact assessment, predicting future diagnosis (Electronic Health Records), biases in training and calibration of AI, explainability and trustworthiness of AI solutions in cancer imaging and polygenic risk score generation and use, as well as clinical application. Key discussion points were collected on a digital mind-map following the ECOUTER stakeholder engagement methodology (11). The results of this co-creative exercise are reported in a separate publication (4). The workshop findings are not presented here as primary empirical data but informed the broader conceptual development of our embedded ethics approach, which is discussed in the final section.

## 3 Findings from the literature review: guiding principles of trustworthy AI

Guiding principles of trustworthy AI have increased in recent years. Building on our previous review (8), this section consolidates the most recent developments around trustworthy AI in radiology, focusing on key dimensions such as explainability and interpretability, trust and trustworthiness, responsibility and accountability, as well as justice and fairness.

### 3.1 Explainability and interpretability

Across the literature, explainability and interpretability are framed as ethical and practical necessities for medical AI, especially radiology. Transparent AI development involving explainability and interpretability is understood a primary ethical concern (12), while at the same time recognizing that ensuring both interpretability and explainability is still an open research area (13).

Ahmed (14) argues from multiple ethical lenses (e.g., fairness, justice, utilitarianism, ethics of care) that opaque black-box models threaten clinicians' ability to judge reliability, with the risks magnified in low-resource settings where second opinions and complementary tools are scarce. Interpretability of AI tools is seen as key to helping clinicians understand and trust model outputs, while black-box models still hinder trust. There is therefore a clear link between explainability and trust across the literature (13, 15–18). Fehr and colleagues (19) run a “reality-check” and conclude that without enforceable, public-facing transparency, black-box AI remains hard to trust or audit in practice, and regulators should mandate substantive disclosure.

Marey and colleagues (20) argue that the limited explainability of modern Machine Learning or Deep Learning can undermine clinician trust, complicate evidence-based decision-making, and raise concerns regarding explainability. De-Giorgio et al. (21) highlight how this opacity makes fully informed consent difficult: patients can't meaningfully consent to tools whose reasoning is inscrutable. For them, transparency is needed to support patient understanding and clinical accountability. There is a risk that lack of explainability and interpretability damages patient-doctor relationships (22).

To assist in attaining a desirable level of interpretability, basic training for radiologists is also seen as crucial, so that they are able to interpret and understand model outputs (23). Ahmed (14) asks for guidance, education, and standards that foreground transparency and bias mitigation, so clinicians and patients can make sense of AI outputs and their limits. Chau et al. (24) consider that this basic explainability and interpretability training should assist clinicians in explaining the potential impact of AI, and that current informed consent practices should be reevaluated to “ensure that patients are fully aware of AI's role, capabilities, and limitations in their care” (24, p. 2), in a way that provides sufficient information about AI. In this sense, “finding the right balance between model complexity, interpretability and transparency is especially important, such that the final performance of the model is not hindered, nor privacy is compromised” (25, p. 263).

The issue of which information is relevant, and for whom, is then crucial. A study on population's perception and knowledge of AI (26) reports that many respondents (i) want to be told if a computer predicts a future disease; (ii) feel they would lack emotional support if results were delivered by a computer; and (iii) have preferences about scan scope (some think AI should only analyze physician-selected body parts; others favor a full-body scan if costs are the same). From the clinicians' perspective, Marey and colleagues (20) report they often want decision-relevant, comprehensible signals (confidence, rationale, case-specific factors) more than a full causal account of the model's inner workings; yet today's systems rarely provide even that consistently. A study (27) examined different opinions from clinicians and technical groups on AI validation in cancer imaging, with technicians valuing most traceability and transparency and clinicians, explainability.

Opacity and how it might hinder access to understanding the tools is linked to epistemic injustice: it affects users in their capacity as knowers. Ahmed (14) highlights epistemic injustice as a core risk in algorithmic radiology. When it comes to testimonial injustice, which can occur when someone is not believed, patients’ reports or experiences may be discounted due to prejudice or baked-in model biases (e.g., dismissing symptoms or signals that don't fit a majority-trained model). Ahmed ties this to under-representation in imaging datasets and to workflow habits that privilege algorithmic output over patient voice. As for hermeneutical injustice, which can happen when someone's experiences are not well understood, communities lack the interpretive resources to make sense of their own experiences (because of language barriers, AI literacy gaps, and inequitable data sharing), which in AI shows up when models and consent materials are built around English-language literature and high-resource settings.

### 3.2 Trust and trustworthiness

Some papers (19, 21, 28–30) link trust with availability of information, while others relate it to robustness (31, 32), transparency (33–35) or fairness (12).

Alvarado highlights that AI systems are, first and foremost, epistemic technologies: information-producing technologies. Therefore, the only appropriate trust AI should receive, if any, is epistemic trust: “the kind of trust that is allocated strictly in virtue of the capacities of the receiver as a provider of information or as a conveyer of knowledge” (28, Introduction). That conceptual move avoids anthropomorphic talk (e.g., care, benevolence) and re-centers the discussion on whether systems are reliable providers of information. Importantly, he adds that even if epistemic trust is the right kind, “whether or not AI can, in fact, be trusted remains an open question,” (28, Abstract).

Bergquist and colleagues (29, p. 346) highlight how trust is a “complex attitude” that involves multiple stakeholders and often leaps of faith rather than absolute certainty; nonetheless this does not mean blind faith, as there are clear aspects that are identified as important to build trust (e.g., reliability, transparency, quality verification, inter-organizational compatibility). In their view, “trust depends on the interaction between the involved parties and should be understood as an ongoing process of establishing faith to reduce complexity” (29, p. 339).

Some authors (36) highlight the importance of honoring patients' autonomy to foster trust, by communicating clearly about data processing; with trust linked to informed consent, transparency in communication, and secure data handling. Fehr et al. (19) analyzed whether current CE-marked AI software systems used in radiology supply enough public information to support those trust conditions, concluding that overall transparency percentages are often very low, with missing documentation on training data, ethical considerations, and limitations for deployment.

The importance of having humans-in-the-loop in order not to corrode trust is highlighted (37), with clear responsibility for diagnosis falling on radiologists and clinicians in order to be able to trust AI in clinical contexts. Nonetheless, Muyskens et al. also argue (37, p. 214) that this general stance might change with time, as development of AI tools progresses, and that generational attitudes with different portrayals of AI in wider media might also influence public opinion.

Providing the perspective of some patients, Fransen et al. (38) survey men undergoing prostate MRI and find high acceptance for AI with a radiologist, modest acceptance for stand-alone AI only if it clearly outperforms clinicians, and a clear intuition about responsibility: hospitals first, then radiologists, then developers. Trust, here, is conditional on both performance and preserved human oversight. Clinician views in Högberg et al. (39) echo that balance. Swedish breast radiologists are broadly positive about AI for workload and sensitivity benefits, yet they flag unresolved risks and, especially, uncertainty over who is liable when AI participates in medical decisions.

Upadhyay (40) reframes trust in operational terms. Although addressing trust hazards such as over-reliance on AI outputs, the author advocates for human-AI teaming with AI as a copilot, so that systems carry information-processing burdens while clinicians retain integrative judgment, ethical reasoning, and communication. Over-reliance and overtrust (37) becomes a greater problem in the case of so-called “hallucinations” (i.e., model confabulations or fabricated outputs without intent), with authors (41) advocating for AI tools to be transparent about their limitations.

The FUTURE-AI guidelines (3) anchor trustworthiness in six core principles (i.e., Fairness, Universality, Traceability, Usability, Robustness, and Explainability, forming the acronym FUTURE) to ensure that AI-tools in healthcare are ethically, socially, medically, and technically sound. The authors argue that trustworthiness is not just about performance, but about multidisciplinary engagement of diverse stakeholders for continuous risk assessment.—AI guidelines

### 3.3 Responsibility and accountability

Across the literature, a strong convergence emerges on the need to locate responsibility in human institutions and agents, and to understand responsibility as a multi-stakeholder process with distributed role-based accountability (14, 29, 38, 39, 42, 43). There are several authors (12, 44) that advocate reconsidering legal frameworks that give all accountability to clinicians when AI systems are becoming increasingly autonomous, and to generate shared accountability frameworks. It is worthy of note that language used to describe human-AI relations can subtly misassign moral responsibility. Metaphors that cast AI as an “assistant” or an “ally” risk suggesting agentic or moral status where none exists, potentially diluting professional accountability or creating misplaced expectations of AI culpability (45). Conceptual clarity is thus crucial to convey the idea that AI is a tool with causal influence but without moral agency.

It is difficult to pin ultimate responsibility on one actor as bias and failure risks are created (and therefore must be owned) across the entire AI lifecycle (46). It is important to explicitly assign roles and responsibilities (18): spelled-out division of labor plus organizational oversight and logs to make accountability actionable (47), clear participant-specific responsibilities for each AI tool (48), and clear lines of accountability (36).

While primary professional responsibility remains with the clinicians and radiologists (14, 49), it must be paired with vendor- and regulator-facing accountability (19). Some papers (31, 32, 42) also frame responsibility as an obligation of health systems and hospitals to institute transparency, governance, and patient-centered oversight before and during deployment, structured disclosure of ethical issues, continuous monitoring and regulatory oversight. Additionally, accountability mechanisms must be anticipatory and structural rather than *post-hoc* fault finding alone (42).

Legal analyses focused on radiology align with this human-anchored approach but sharpen it into attribution rules for harm. Clinicians must either reasonably rely on or defensibly depart from an AI system's output according to prevailing standards of care (49). Crucially, responsible use presupposes that clinicians understand AI performance characteristics and communicate AI's role to patients, requirements that turn explainability and documentation into prerequisites of accountability in clinical practice (30). As no AI-specific health legislation presently governs radiology, accountability must be constructed from general legal and ethical principles such as duties of care, informed consent, device regulation (50).

### 3.4 Justice and fairness

Some authors (29, 51) advocate a procedural fairness that highlights the need for transparency, traceability, and local validation to enable just use. Additionally, the literature also shows accounts of fairness as related to issues of distribution involving access, representation, and results (14, 47). According to this account, AI's benefits must reach under-resourced settings and under-represented groups. Fairness is understood not merely model performance parity but addressing global and local access gaps, and there is concern about equitable access and who can afford advanced medical technologies (16, 36).

In this sense, some (43, 46) consider that fairness must be both measured and, also, socially grounded. In particular, Gichoya and colleagues (46) warn that metric-only fairness can miss systemic inequities and hidden signals. Some authors (12, 41) consider that a social justice framework is needed, so that fairness is embedded in AI with democratic and justice-oriented principles.

Bias appears also considered as linked to dataset heterogeneity and demographic representativeness (15, 17, 18) which can limit generalization (33) and trust (3). In the case of gender bias, this gender representativeness can be embedded in the way algorithms are trained: “many cardiovascular risk prediction algorithms have a history of being trained primarily on male patient data. This has led to an inaccurate risk assessment in female patients with different symptoms and risk factors” (47, p. 5). Some recommend reporting the performance of their methods under diverse racial and gender contexts, “to ensure the advancement of fair and trustworthy AI, devoid of racial, social, and gender biases” (12, p. 14).

When it comes to understanding of fairness and expectations of different actors, some report lay expectations from patients that hospitals, developers and clinicians collectively prevent unjust harm (38). From the perspective of clinicians, Högberg et al. (39) documents their insistence on standards that equally distribute benefits and harm, and curtail bias in screening contexts that affect large populations.

Measures to contribute to justice and fairness call for global initiatives to ensure equal access (14); demands of diverse teams, representative data, and contextual validation (46), the need of transparency, traceability, and local validation (29), paired with guidance and training to mitigate bias and black-box opacity (51). Fairness requires diverse teams, context-aware validation, and monitoring to avoid the danger of replicating systemic biases (36, 37, 46). In order to mitigate bias, the FUTURE-AI guidelines recommend not assuming sources of bias and instead engaging in a multistakeholder process to “form interdisciplinary AI development teams and investigate in-depth the application specific sources of bias, which might include domain specific attributes (e.g., breast density for AI applications in breast cancer)” (3, pp. 13–14).

While the literature distinguishes between several guiding dimensions of trustworthy AI—explainability and interpretability, trust and trustworthiness, responsibility and accountability, and justice and fairness—these dimensions are analytically separable but empirically intertwined. Notably, trust and trustworthiness function as integrative concepts through which the other dimensions become meaningful in practice. Explainability, fairness, and accountability are often discussed in the literature as preconditions for trust. For this reason, in the empirical section we pay particular attention to how trust and trustworthiness are constructed, negotiated, and experienced in real-world radiological contexts, while remaining attentive to their connections with the other dimensions.

## 4 Empirical findings

As we highlighted in our literature reviews, the concept of trustworthiness has become a defining condition in the development and application of ethical AI. Whereas social scientific perspectives describe trust as fundamental for interpersonal interactions and society at large, approaches on trustworthy AI are addressing trust in AI mainly in terms of technical conditions and solutions. The social character of trust has received rather little attention and thus the interpersonal relationships, institutional conditions, and societal contexts that are of particular importance in health systems and settings. Trust plays a central role in situations of risk, vulnerability, and uncertainty; situations that are often encountered in the medical context. Sociological approaches claim that trusting relationships and trust in an institution, e.g., the healthcare system, is determined by the trust in its representatives, e.g., doctors, and, vice versa, trust in the medical system provides the legitimacy of its experts in providing a trustworthy environment (52). To interpret how stakeholders describe trust in practice, we draw on sociological approaches that conceptualize trust as relational, processual, and situated within institutional contexts. These theoretical perspectives are not introduced to redefine the study's focus, but to analytically deepen the interpretation of the empirical material.

In following the concept of trust that is constituted by several trustor/trustee relationships, such as trust in persons (e.g., scientists who trust each other, patients who trust scientists and clinicians), technology, and institutions (53, 54), we identified several understandings of trust in/trustworthiness of AI in our empirical study, which we will highlight in the next sections.

Consistent with the findings from our literature review, the empirical material also highlights that trust and trustworthiness are frequently linked to the explainability and interpretability of AI systems. However, the emphasis is placed less on understanding the technical inner workings of an AI model and more on knowing the criteria it uses to reach a decision. The type and depth of knowledge considered necessary for fostering trust varies depending on who the users are, and what level of detail is meaningful for their roles. As one interviewee noted, radiologists and “people in the middle” (such as radiographers) require a practical yet critical understanding of the system: one that is sufficiently detailed to support their everyday clinical decision-making without demanding full technical expertise.

“[…] maybe you should be aware of, you know, how the decisions are made at least. So you will be able to explain it to a patient or at least to detect when something is wrong, right? […] I think it's quite interesting to sort of like provide, you know, […] explaining what it does. […] And also it's very important to say that the […] end user shouldn't trust 100% the results. […] And actually the AI results should be accompanied with some explanation. And here we talk about explainability. You know, to make sure the decision is sort of like a backup with something that makes sense. Because it has been in many cases that software got the right answer, but because of the wrong sort of like a purpose.” Engineer

The understanding of how the system works does not necessarily require additional qualification by medical professionals but can be arranged in interdisciplinary institutional arrangements, as the following interview partner highlights:

“[…] for implementing these tools, for trusting them, if we don't understand something to have someone that may go to the code and let the physician know why it's working like this, or if it doesn't work, what's going on, we need also engineers in the radiology departments.” Academic radiologist

As shown in the reviewed scientific literature, the concept of trust is frequently conflated with related terms such as acceptance and explainability, where explainability itself is often intertwined with notions of transparency, accountability, and reliability. These terms are commonly used without clear definitions or explicit delineation of their conceptual boundaries. Our empirical material indicates that professionals similarly employ a range of terms—such as accuracy or credibility—to describe their expectations of AI systems:

“I’m curious to know what is the accuracy of the system.” Radiologist

“[…] trust in AI directly relates to credibility of AI tools. […] it means that I can depend on the tool and be sure that it will do exactly what was promised by the AI developers.” Clinician

This conceptual variety reflects broader expectations towards explainability while still pointing to a shared underlying intention. Rather than full access to the technical “black box” of AI decision-making, professionals primarily expect the decision process to be traceable. Such traceability serves as evidence that the AI system is trustworthy as a tool, fulfils its intended purpose, and produces outcomes that are intelligible to medical professionals. This is illustrated in the following quotes:

“The problem of this approach using data-driven models, and algorithms in research, is that usually our systems that cannot be explained by themselves. There is no explanation about the results. Probably there, if the process is well done, probably you would achieve a good performance of the algorithm in place, but you don't know the reasons why the algorithms propose this one thing or another. This is one important point, no? And medical science is based on trust. And if they cannot explain what's the reason of the recommendations, the trust can be put to doubt.” Physician

“If I were a doctor, I would rather have a model that gives a confidence interval. Just instead of only giving a behind the scenes. Also catching all the errors and mistakes, all the adversarial attacks. If you can detect it, of course it builds more trust.” Engineer

Trust in AI is trust “in the making”. It is not established through abstract definitions or technical explanations alone but emerges gradually through repeated interactions with the system. To be convinced of an AI system's accuracy and usefulness, professionals must accumulate experiences that validate its performance within their specific clinical context. This experiential dimension of trust-building complements the broader expectations of traceability and intelligibility: while clear and comprehensible decision pathways provide an initial foundation, sustained trust develops when the system consistently demonstrates reliability in practice. In this sense, trust is not a static prerequisite, but an evolving process shaped by ongoing engagement, observation, and professional judgement.

“The doctors still trust the imaging, right? But there is no way of validating that these images are actually from what is inside the human body. Why do they still trust? Why do they trust scanners? […] For scanners, maybe in the beginning people were not trusting when the MRI scans came. […] Now we cannot live without it. AI is going to become like that, right? In the beginning nobody trusted. All these mechanisms are added on top of it, explainability, interpretability, uncertainty estimations. Maybe we will get there when they start trusting AI the same way they trust now the MRI scanners. It takes time.” Data scientist

As mentioned earlier, trust is situated at the intersection between individual and system. Empirical finding show that perceptions of an AI application's trustworthiness may be shaped by several reference points: the professional community, e.g., the expectation that an AI system performs as good as an “average radiologist”; the scientific domain, e.g., where trust is reinforced through evidence published in high-impact journals demonstrating the system's accuracy; and, finally, trust is also informed by the expertise and judgement of individual practitioners, who compare their own interpretations of an image with the outputs generated by an AI system. Moreover, trust in or trustworthiness of AI is situated within the broader tension between scientific practice (grounded in rational reasoning, empirical validation, and evidence-based evaluation) and the inherent uncertainty that characterizes medical decision-making. In this context, “gut feelings” often serve as discursive proxy for the emotional and intuitive dimensions of clinical judgement. These affective elements play a crucial role in how professionals assess and rely upon AI systems, highlighting that trustworthy AI cannot be conceptualized solely in technical terms but must also account for the experiential and emotional realities of medical practice.

“I just know about the feelings of not feeling confident about using something. […] Sometimes many things in medicine have happened almost by gut feelings.” Academic radiologist

Gut feelings or intuition are informed by past experiences and, as has also been shown in our previous research (55), are also essential for research participants and patients in making decisions. In this context, the human factor is particularly evident as trust is needed to manage uncertainty—and this also refers to the accountability of human actors in dealing with the uncertainty that AI might create and the responsibility that comes with it:

“The problem is that the input we are going to enter is very subjective. The final diagnosis is also subject to subjectivity, so we don't have 100% guarantee that the final diagnosis is correct. […] So everything is very subjective. So we are turning a system with subjectivity. […] essentially nobody will take responsibility of a software. If a software is wrong, who is responsible for this failure? I prefer to assume my risks, assuming that I’m a human and my opinion may be wrong, then delegating my responsibility to a software. Essentially, this is the main problem of AI.” Radiologist

The agency attributed the medical profession in its interaction with the machine mentioned therein is likewise reflected in how patients understand the role of AI in clinical practice.

“I mean, imaging has changed the paradigm of diagnosis. Starting from mammography and MRIs and- Who can deny that? So if we can add something to help the physicians, so if the machines can learn from many cases, and that is the database purpose, where the lesion lays, it's interesting. But should we leave the machine performing by itself? I don't think so.” Patient representative

A patient representative provides a clear illustration of how patients, doctors, and AI systems may interact in practice, highlighting the dynamic interplay of responsibilities, expectations, and agency among these actors:

“I would like to see a very strong AI capable of spotting illness where the doctors cannot see. Very powerful tools but guided by doctors. With the clinician eye between the results and the patient.” Patient representative

From a sociological perspective, trust plays a crucial role in managing uncertainty and knowledge gaps. Hence, the relationship between trust and uncertainty in the context of AI needs careful examination. Within this conceptual framework, the assumption that increased knowledge automatically leads to greater trust is misleading, since complete knowledge would make trust unnecessary in the first place. Our empirical findings suggest that what matters instead is an awareness of uncertainty and a nuanced understanding of which forms of knowledge are meaningful for which users. Such differentiation is essential for fostering professional agency and ensuring accountability in the use of AI systems.

## 5 Discussion: future directions—towards embedded ethics and SSH research in medical AI

The Discussion moves from results to implications in two steps: first, we synthesize recurring empirical patterns from the interviews (e.g., trust as relational and practice-based; accountability as distributed; explainability as role-dependent and workflow-bound); second, we interpret these patterns together with the literature review to derive governance-relevant implications. On this basis, we propose embedded ethics/SSH involvement as a practical way to operationalize trustworthiness across the AI lifecycle.

The findings of our study support that trust in radiological AI develops within multilayered contexts and not solely through sorting out technical aspects. This view often obscures the basic need to examine the socio-technical environments in which AI is built, deployed, and justified as a source of knowledge, as an epistemic technology that provides of information and enhances our capacities as knowers (28). The confidence an epistemic community places in science, and in the information produced or shaped by AI, rests not only on technical performance but also on the ways these systems are embedded within institutions, practices, and prevailing norms (56, p. 20; 57, p. i). Additionally, even some aspects that are usually treated as technical have ethical and societal dimensions (e.g., transparency also has to do with epistemic justice, which respects our right to be informed about technology that is going to affect our lives, and with the transparency of institutions).

The empirical data indicated that trust is something that grows through experience, comparison with human judgments, and the consistent performance of AI tools in clinical contexts. Trust depends on whether AI supports clinical reasoning, fits local workflows, and aligns with established professional relationships. To be able to understand the nuances of these multilayered contexts, it is crucial to conduct interdisciplinary work that includes medical professionals, engineers and data scientists, and also experts in SSH, who have the expertise to understand broader ethical and societal aspects that also must be considered. More SSH research is needed nowadays to understand the complexity of these tools' impact, which goes beyond technical aspects.

In our previous review (8), we had already identified the lack of analysis of the broader societal origins of bias. Bias was often treated as a technical problem with a technical solution. Thus, we advocated a strong integration of a SSH perspective into interdisciplinary research, that could delve deep into the sociotechnical aspects of bias in AI models in medicine. In this paper, we must advocate it again. While recent literature, published in the last 2 years, mention larger sociotechnical contexts when it comes to perpetuating pre-existing biases (14), or highlight the need of diverse stakeholders in developing AI solutions (3, 29, 43), there persists a lack of SSH analysis. Interdisciplinarity is particularly understood as bridging the gap between technical, legal and clinical perspectives (27, 50, 58). Only two papers (28, 45) conduct a deep SSH analysis of certain aspects of AI in radiology.

There is therefore a need for embedding ethics and SSH research as integral parts of medical AI development and deployment. Some authors have proposed what they have called translational medical humanities (59) or embedded ethics (60, 61). A translational medical humanities approach considers the humanities not only as auxiliary to medical research and practice, and as an approach creates “an interdisciplinary space where both medicine and the humanities challenge and inform each other across the mega-divide between the humanities and the natural sciences” (59), Introduction). Embedded ethics refers to the integration of ethical considerations into the design and development process of technology (60, 61). It involves identifying potential ethical issues and addressing them proactively to minimize harm and promote ethical behavior. In this sense, it is important to introduce interdisciplinary knowledge of societal issues from the beginning of the development of AI applications and throughout all stages of design, development, and implementation.

Our findings indicate that trustworthiness in radiological AI is shaped by relational, institutional, and socio-technical dynamics that extend beyond technical performance alone. While our data do not “prove” that embedded ethics is the only possible governance approach, they consistently highlight challenges—such as distributed accountability, context-dependent intelligibility, and socially embedded bias—that are difficult to address through principle-level guidance alone. In light of these patterns, we argue that embedding ethical and SSH expertise throughout the AI lifecycle offers a coherent and practically responsive way to operationalize trustworthiness in clinical settings.

The case of sex and gender bias can be illustrative of how an interdisciplinary collaboration that includes SSH researchers can lead to fruitful exchanges. Biases are not a mere artifact of flawed code: they are the predictable result of training algorithms on datasets created within unequal societies (62–68). Technical fixes alone cannot surface the deeper assumptions about whose experiences are centered, whose bodies are considered standard, and whose lives are made legible to the system (69). Understanding how sex and gender biases infiltrate medical AI demands literacy in the social and historical dynamics through which sex and gender are constructed, enforced, and measured. Scholars in gender studies have long shown that categories like “man” or “woman”, and even “male” and “female”, are not neutral descriptors but institutionalized frameworks shaped by power, exclusion, and cultural norms (70–73). Interdisciplinary teams that include gender-theory scholars can identify deeper mechanisms of bias earlier, question the tacit assumptions built into datasets and labels, and guide the creation of medical AI that is more accountable and inclusive.

Nonetheless, “currently, the people who are reading gender theory are not the co-authors of papers such as ‘Turning a blind eye: Explicit Removal of Biases and Variation from Deep Neural Networks (Alvi et al., 2018)’” (74, p. 10). From our sample, in more recent literature, there is a general understanding of the importance of not discriminated against vulnerable or historically underserved groups, but without consideration of what the societal at play are, and how they might affect datasets, scientists” biases, or institutional dynamics. The integration of SSH perspectives might shed light on deeper ways to address and correct bias.

The results of our research indicate that it is important to pay close attention to potential biases in the training and validation data of AI to produce fair algorithms, in particular to sex and gender dimensions in datasets, rather than ignoring them (75). Some researchers (76, 77) suggest introducing a desirable bias to counteract the effects of undesirable biases that can lead to unintended or unnecessary discrimination. Moreover, it is important to challenge background assumptions (78, p. 60) about sex and gender to see how these might affect scientific practices (79–82).

It is often assumed that data is objective and neutral, but data is always embedded in a particular context (83, 84). This means that data is always influenced by the assumptions and biases of the person or group collecting and analyzing the data. Therefore, it is crucial to question these background assumptions and consider the context in which the data was collected to arrive at a more accurate and nuanced understanding of the data. It is important to pay attention to the specific context of application and the specific group attributes of the population where the AI tool is applied. Attention to context, including a deep understanding of pre-existing inequalities and vulnerable groups, is also paramount to implement AI in real-world situations.

Findings reveal several reference points when estimating AI trustworthiness, such as professional community norms, scientific validation, and personal (and others) expertise, emphasizing that trustworthiness is co-constructed across these levels. Hence, assuming that more knowledge increases trust is overly simplistic. Empirical findings challenge deterministic or purely technological accounts of trustworthiness and offer a human-centered understanding of how AI is experienced in practice. Understandings of and trust in AI develops over time through interactions and accumulated experience, underlined by conceptualizations of trust as “in the making” and aligns with sociological approaches that view trust as relational, contextual, and practice-based. Consequently, trust evolves through use, not prior to it, and trust should be analyzed as a socio-material process rather than a user attitude.

As supported by the empirical data, doctor-patient relationships are crucial when it comes to trust; there are trust relationships that have already been established and even institutionalized and AI applications need to build on these relationships and fit into these institutional dynamics. Trust needs to be understood within the described relational configuration, highlighting the socio-technical nature of AI integration in clinical practice. To do that, it is important to assess different specific factors that may affect the AI tool's performance in real-world situations. If an AI tool is trained on data that is representative of the real-world variations encountered in clinical practice, it is more probable that clinicians, citizens, and other stakeholders will have trust in its use.

Keeping humans in the loop or proposing an approach that privileges human oversight appears paramount to building trust in AI applications. However, human oversight alone is insufficient if clinicians lack adequate understanding of AI outputs, explainability tools, or cognitive vulnerabilities such as automation bias. Without appropriate education and critical AI literacy, the human in the loop may inadvertently reinforce rather than mitigate errors. Furthermore, to ensure that the development and implementation of AI technologies are effective and beneficial, it is crucial to define the relevant stakeholders and involve them from the early stages of the research process. This includes interdisciplinary teams, patients, and other healthcare professionals. Meaningful involvement is key, which means creating a space for stakeholders to interact with each other and engage in knowledge-building exercises. It is important to define what needs to be known by different stakeholders. It is often stated that for trust to be gained, there should be extensive knowledge about the inner workings of algorithms and AI applications, and that the existence of more data will lead to more trust. Nonetheless, it is a matter of engaging relevant stakeholders in knowledge-building exercises in which information can be shared, and needs can be identified. What is relevant for different stakeholders is context- and user-dependent. By involving all relevant stakeholders in decision-making, we can ensure that AI technologies are developed and implemented in ways that are both effective and ethical.

These insights underline the need to embed SSH perspectives throughout the entire AI lifecycle (see Table 1). Interdisciplinary and ongoing engagement (including clinicians, patients, ethicists, social scientists, developers) support ensuring that intended uses, forms of explainability, and accountability requirements are defined early and refined continuously, as well as identifying broader sources biases and potential social impacts, and tailoring systems that fit the realities of radiological work.

**Table 1:** Empirical patterns and implications.

Empirical pattern	Implication
Stakeholders describe trust as “in the making” through experience and institutional arrangements	Trustworthiness requires ongoing socio-technical governance, not only technical performance claims.
Explainability is framed as decision-relevant intelligibility rather than access to model internals	Interdisciplinary translation work is needed (clinical, technical, SSH) to define “meaningful” explanations for different roles
Responsibility is discussed as unavoidable and human-anchored yet practically distributed	Need for role clarification, accountability mechanisms, and documentation practices that are organizational as well as technical
Bias/fairness concerns point beyond metrics toward context and power relations	Value of SSH expertise to surface assumptions and impacts early and continuously

## 6 Recommendations for future practice

Based on an embedded ethics approach (61) and our work on multi-stakeholder involvement in the development of trustworthy AI (4), as well as the findings from our empirical study, we suggest the following structured AI development pathway:

### 6.1 AI design phase

Involving stakeholders, such as clinicians, patients, ethicists, and social scientists from the outset helps define intended use, transparency needs, and potential harms and ethical risks. This ensures that social implications and contextual factors that shape how AI systems are perceived and used are considered, and that trustworthiness is co-designed in a situated manner.

### 6.2 AI development phase

Stakeholders provide valuable feedback on datasets, model architecture, user interfaces, and workflow integration, while ethical engagement supports the interpretation of ethical principles, analyze emerging issues, and support justifiable decision-making. The enables translating trustworthy AI principles into practice by embedding bias mitigation, transparency approaches, usability considerations, and human oversight directly into model and system design. Users, including radiologists and clinicians, must be able to understand the criteria that guide AI in decision-making to attain a certain level of intelligibility of the system to effectively integrate it into their daily clinical practice. To realize this, interdisciplinary institutional arrangements are needed as well as traceable, intelligible AI decision-making.

### 6.3 AI validation process

Validation requires assessing robustness, fairness, and explainability in real-world conditions. Usability assessments enhance workflow fit, while ethicists and social perspectives assess relational and institutional dynamics. This ensures that trustworthy AI is not only defined by technical metrics, but also in relation to human decision-making, doctor-patient relationships, and equitable care. In line with broader methodological critiques of single-metric evaluation frameworks in AI research, trustworthiness cannot be reduced to isolated quantitative indicators; rather, there is need for problem-aware selection of multiple complementary metrics (85). This represents “trust in the making” during which professionals must accumulate experiences that validate the AI systems performance within their clinical context and provides the space for trust as an evolving process shaped by ongoing engagement, observation, and professional judgement, allowing professionals to compare their interpretations of an image with the outputs generated by an AI system.

### 6.4 AI deployment phase

Monitoring the systems' performance and impact in real-world settings with clinicians, patients, developers, ethicists, and social scientists supports continuous evaluation of performance and emerging ethical risks. Embedded ethics emphasizes transparent documentation, accountability mechanisms, and governance structures that prevent “ethics washing” (86) and uphold ongoing trust, safety, and human oversight.

Embedding ethics and SSH research together with multi-stakeholder involvement enables a continuous, iterative, and collaborative process that reflects the core dimensions of trustworthy AI—safety, fairness, transparency, accountability, and human-centeredness across the entire AI lifecycle. It transforms abstract ethical principles into practical development practice, ensuring that medical AI remains trustworthy in design, deployment, and real-world use.

## 7 Conclusions

In our literature review, we highlighted key guiding principles of trustworthy AI in the current scientific discourse: explainability and interpretability, trust and trustworthiness, responsibility and accountability, and justice and fairness. While all are recognized as essential for responsible AI deployment, they are difficult to achieve in practice without more concrete steps on how to move beyond high-level principles, especially how to embed ethics and involve stakeholders throughout the entire AI lifecycle.

By combining an embedded ethics approach (61) with or previous work on multi-stakeholder involvement in the development of trustworthy AI (4), and integrating insights gained from our empirical study, we proposed a structured AI development pathway. It emphasizes continuous, interdisciplinary engagement to ensure that ethical principles and trustworthiness requirements are integrated in practice.

## Data Availability

The datasets presented in this article are not readily available because the data underlying this study represent personal opinions and practical experiences of professionals active in a very specific field. Since the specific roles and frameworks in combination with the sample size would likely allow identification of the participants, the dataset cannot be shared publicly. Additionally, the Research Ethics Committee of the Medical University of Graz has determined that the data should be thoroughly deidentified. Requests to access the datasets should be directed to Melanie Goisauf, melanie.goisauf@bbmri-eric.eu.

## Supplementary material

Supplementary File S1 Results of the updated literature review in an adapted PRISMA flow diagram (Page et al., 2021).

Supplementary File S2 Search strings, databases, and outputs of the rapid literature review.
