# Supplementary material for: Beyond the algorithm: embedding ethics for trustworthy AI in radiology and oncology
Source: Front Digit Health. 2026 Apr 20;8:1756256. doi: 10.3389/fdgth.2026.1756256 (PMC13136111; doi:10.3389/fdgth.2026.1756256)
Supplement: Supplementary File S1 — Results of the updated literature review in an adapted PRISMA flow diagram (Page et al., 2021). [file Supplementaryfile1.pdf]

frontiers

Supplementary Material

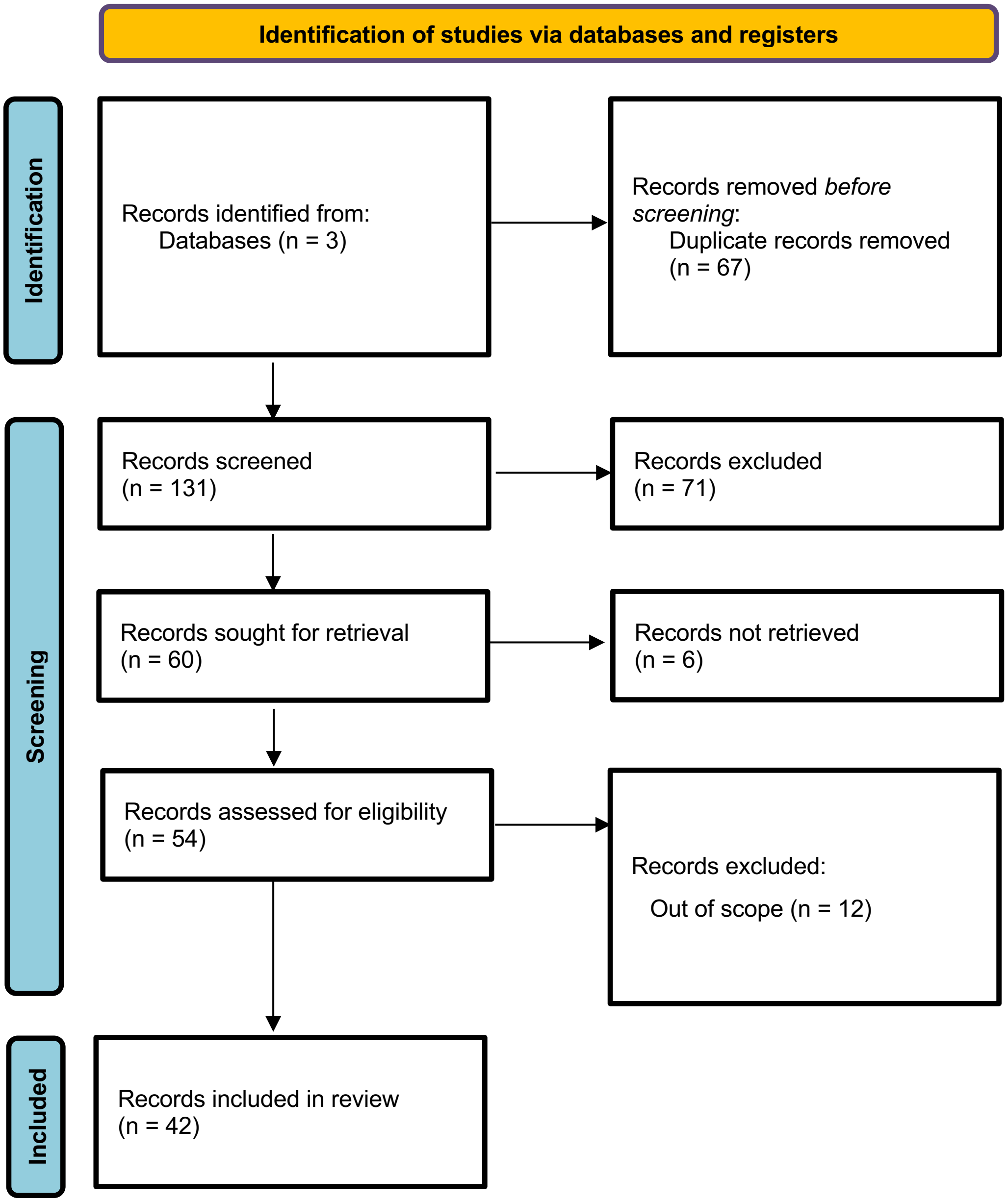

Supplementary Figure 1. Results of the updated literature review in an adapted PRISMA flow diagram (Page et al., 2021)
