# Supplementary material for: Beyond the algorithm: embedding ethics for trustworthy AI in radiology and oncology
Source: Front Digit Health. 2026 Apr 20;8:1756256. doi: 10.3389/fdgth.2026.1756256 (PMC13136111; doi:10.3389/fdgth.2026.1756256)
Supplement: Supplementary File S2 — Search strings, databases, and outputs of the rapid literature review. [file Supplementaryfile2.pdf]

Supplementary Material

	Search results by database		
	Google Scholar[1]	*PubMed*[1]	*Scopus*
((AI) AND (ethics)) AND (radiology)	10	10	10
(((black box) AND (AI)) AND (ethics)) AND (radiology)	10	10	3
(((bias) AND (AI)) AND (ethics)) AND (radiology)	10	10	9
(((Trust) AND (AI)) AND (ethics)) AND (radiology)	10	10	6
(((trustworthy) AND (AI)) AND (ethics)) AND (radiology)	10	10	1
(((explainability) AND (AI)) AND (ethics)) AND (radiology)	10	10	3
(((interpretability) AND (AI)) AND (ethics)) AND (radiology)	10	10	1
(((responsibility) AND (AI)) AND (ethics)) AND (radiology)	10	10	4
[1] limited to 10			

Table 1 Search strings, databases, and outputs of the rapid literature review
